# Transoral Robotic Surgery for Oropharyngeal and Hypopharyngeal Squamous Cell Carcinoma

**DOI:** 10.7759/cureus.57186

**Published:** 2024-03-29

**Authors:** Wilhelmina Tan, Rebecca Bui, Viran J Ranasinghe, Orly Coblens, Sepehr Shabani

**Affiliations:** 1 Otolaryngology - Head and Neck Surgery, University of Texas Medical Branch (UTMB), Galveston, USA

**Keywords:** functional and clinical outcome, head and neck neoplasms, hypopharyngeal cancer, oropharyngeal scc, transoral robotic surgery

## Abstract

With oropharyngeal cancer incidence rising globally, largely due to human papillomavirus (HPV), and hypopharyngeal cancer known for poor outcomes, innovative treatments are needed. Transoral robotic surgery (TORS) offers a minimally invasive approach that may improve upon traditional open surgery and radiotherapy/chemoradiotherapy (RT/CRT) methods.

We conducted a literature review and included 40 PubMed studies comparing TORS, open surgery, and RT/CRT for oropharyngeal and hypopharyngeal squamous cell carcinoma (SCC), focusing on survival rates and swallowing function outcomes.

TORS provides favorable survival outcomes and typically results in superior swallowing function post-treatment compared to other therapeutic modalities in both oropharyngeal and hypopharyngeal SCCs.

The clinical benefits of TORS, including improved operative precision and minimized tissue disruption, along with the elimination of surgical incision recovery and reduced RT toxicity, suggest it is a valuable surgical approach for head and neck cancers.

## Introduction and background

The rise of surgical robotic technology for head and neck tumors offers an alternative to traditional open surgical approaches. While the traditional invasive surgical approach allows for a wide surgical field for better visualization and ease of tumor resection, it is often associated with a long-term sequela of poor quality of life factors including impaired swallowing functionality postoperatively [1]. Transoral robotic surgery (TORS) is a minimally invasive, robotic surgery technique that can be used to remove tumors in the oropharynx and hypopharynx. Since its 2005 clinical debut in head and neck surgery, TORS has shown promising results due to enhanced visualization, precision, and efficiency [1,2]. With the introduction of the Single-Port (SP) da Vinci Robotic Surgical System by Intuitive, TORS routinely utilizes two arms and a camera through a single port. One arm is equipped with a high-resolution camera that provides an optimized, unobstructed three-dimensional view of the operative field via a computer console. The other two side arms serve as working "hands" of the robot that mimic a surgeon's arm and hand movements with an improved range of motion carrying miniaturized tools at the end of interchangeable instruments. The control configuration system delivers full-wristed articulation which parallels the surgeon's wrist, hand, and finger movements to direct precise fine motor movements scaled down to size for complete operative control with tremor filtration, thereby resulting in less disruption to nearby tissue in deep anatomical spaces [3].

In addition to TORS and traditional open resection, therapeutic modalities used in the management of pharyngeal carcinomas include nonsurgical therapy which encompasses radiation therapy (RT) and chemoradiotherapy (CRT). 

While TORS is increasingly adopted, comprehensive comparisons with traditional methods regarding long-term outcomes like quality of life and functional preservation remain underexplored. This study aims to bridge this knowledge gap by evaluating survival rates and swallowing functionality across clinical cases managed by open resection, TORS, or RT/CRT as carcinoma management therapies in the oropharynx and hypopharynx. Given the potential of TORS to reduce postoperative morbidity, our findings shed some light on the usage of TORS and the management of pharyngeal carcinomas by prioritizing both oncologic control and quality of life.

## Review

The global rise of oropharyngeal cancer incidence is primarily attributed to the increased human papillomavirus (HPV) infection [4]. HPV-positive oropharyngeal cancers have risen by 225% from 1988 to 2004, and in 2010, HPV-positive tumors made up 63.8% of all oropharyngeal cancer cases [5,6]. Compared to pre-1995, by 2014, the rate of HPV-positive oropharyngeal cancer increased by 20.6% worldwide [7]. In France alone, from 2011 to 2021, the prevalence of HPV infection increased from 43% to 57.3% in oropharyngeal cancer [8]. Modern management of oropharyngeal carcinomas favors minimally invasive surgery over traditional open resection to potentially reduce morbidity and improve functional outcomes including swallowing functions or tolerance of oral diet vs feeding tube dependence. However, continued research into oropharyngeal management strategies is critical in assessing and matching patients to the most appropriate treatment options [9].

Evidence supports the viability of TORS for patients with oropharyngeal cancer, demonstrating safe implementation without necessitating conversion to open surgery, and many consistently demonstrated negative margins in TORS. A cohort led by Benazzo et al. reported no positive margins in all TORS cases with no intraoperative adverse events and no conversion to open surgery. It was also reported that TORS showed a quicker recovery time, and no recurrence of malignancy was reported by the time the study was submitted [10]. A Japanese center noted the successful completion of TORS in all eligible patients with T1-T3 oropharyngeal cancers without needing to be converted to open cases [11]. Similarly, findings from Rubek et al. in Denmark demonstrated the safety of TORS for achieving negative margins in 96.7% of patients with T1-T2, N0-N1 oropharyngeal cancer [12]. In a prospective study done in the United States with 20 patients, no intraoperative or postoperative complications were reported in any patients undergoing TORS [13]. Collective data from various international studies affirm the safety and feasibility of TORS as being an alternative to the traditional treatment modalities in oropharyngeal cancers with minimal conversion rates and rare intraoperative or postoperative complications.

Preliminary data for TORS survival and functional outcomes is also promising. A 2010 study by Weinstein et al. demonstrated a disease-specific survival rate of 98% in one year and 90% in two years following TORS [14]. Similar data was produced by Cohen et al. with the disease-specific survival at one and two years 97.8% and 92.6%, respectively. Overall survival within the same cohort of patients was 95.7% at one year and 80.6% at two years following TORS [15]. A 2010 study by White et al. revealed the two-year recurrence-free survival rate for a cohort of 71 patients was 86.5% for patients treated with TORS [16]. This is echoed by van Loon et al., in their 2015 study done in the Netherlands, where 88.9% of patients with T1-T2 oropharyngeal carcinoma did not have a recurrence of cancer post-TORS during the long-term follow-up and reported having good quality of life based on surveys [17]. Together, these studies suggest that TORS offers favorable survival outcomes for early-stage oropharyngeal carcinoma.

Swallowing is a functional outcome that is a significant quality of life factor used to evaluate TORS. Acute post-TORS dysphagia is a common complication. The dependence on a feeding tube suggests poor swallowing functionality. Early in TORS postoperative periods, patients experience declines in eating quality of life domains with 24% of patients requiring gastrostomy tubes at six months and 9% at one year [18]. In a study done by Feng et al., the feeding tube rate at some point during treatment following TORS for oropharyngeal squamous cell carcinoma (SCC) was 8% (11 patients out of 138) with only 0.7% of patients (one patient out of 138) being gastrostomy dependent after one year [19]. In a case series done in an academic hospital by Iseli et al., 9.5% had retained feeding tubes, and 6% had aspiration with a mean follow-up duration of 12 months [20]. Research done by Park et al. demonstrated an oral diet was tolerable after a mean of six days following TORS with 97% of patients swallowing well [21]. Weinstein et al. reported one patient required a gastrostomy tube one year after TORS treatment [14]. Findings by White et al. suggested that at the last follow-up (median 26 months), all patients regained full swallowing ability with no patients dependent on a feeding tube [16]. Hans et al. found that in their investigation, patients receiving TORS with or without adjuvant CRT had no need for a feeding tube or tracheostomy and all had reported satisfactory swallowing quality, although patients receiving TORS alone had better functional outcomes than patients receiving both TORS and adjuvant CRT [22]. Ongoing long-term functional outcomes improve with time across multiple studies, indicating that favorable swallowing results within one year following TORS may outweigh the acute side effects and complications.

In a study comparing TORS versus intensity-modulated radiation therapy (IMRT), a type of radiotherapy aimed at increasing the accuracy of radiation to its target and decreasing radiation dose to bystander structures, Yeh et al. found comparable survival outcomes. However, selection bias must be considered as TORS studies included patients with an earlier stage of oropharyngeal SCC compared to patients in the IMRT studies [23]. de Almeida et al. found similar survival rates as well for patients undergoing TORS or IMRT. The two-year overall survival rate is estimated between 84% and 96% for IMRT and 82% and 94% for TORS. Some patients in the IMRT studies also received CRT and neck dissections, while some patients in the TORS studies received adjuvant RT or CRT which influenced the survival rates. de Almeida et al. recorded adverse events and showed that the use of a gastrostomy tube was less in TORS [24]. In the phase 2 trial of a recent study, Nichols et al. found no clinically meaningful change in swallowing function after one year of treatment between patients treated with TORS or radiotherapy [25]. In the long-term follow-up of the trial that Nichols et al. conducted, beyond one year, the swallowing quality of life differences between RT and TORS got smaller, and the initial pain and dental concerns that were more prominent in TORS compared to RT were in fact resolved beyond one year, and dry mouth remained more persistent and prominent in RT patients [26]. An investigative effort by More et al. concluded that in patients with stage III or IVA oropharynx and supraglottis SCC, while TORS with adjuvant therapy group did not show a difference in swallowing functions before treatment or three months post-treatment compared to the CRT group, differences start to show past that time point. At half-year and full-year follow-up times, the swallowing function of the TORS group was significantly superior to the CRT group [27]. These studies suggest the survival rate between TORS and RT may be similar, while the functional outcome of swallowing is comparable or superior in TORS than in RT. 

In a comparative study between TORS and another nonsurgical treatment modality, CRT, Meccariello et al. found no statistically significant difference in the five-year overall survival, disease-free survival, local recurrence-free survival, and regional recurrence-free survival. Low feeding tube dependency was noted for TORS compared to the CRT group with 1.7% and 4.8%, respectively [28]. Sharma et al. also showed no statistically significant difference in survival between TORS and CRT groups. However, the feeding tube rate between the groups was remarkably different. Around 33.3% of patients in the TORS group had a gastrostomy tube placed after treatment, while the rate was 84.1% in the CRT group with both groups having a substantial reduction in gastrostomy tube prevalence at the three-month and 12-month markers [29]. TORS patients in a study by Genden et al. had better short-term eating ability two weeks after treatment compared to the CRT group, further suggesting CRT is associated with worse swallowing function [30]. Compared with CRT, TORS was found to have a significantly higher survival at three years (83% vs 57%), in a study done by Smith et al. Additionally, it was found that TORS plus neck dissection was sufficient for accurately staging oropharyngeal carcinomas to determine appropriate therapy options [31]. In conclusion, survival rates are similar between TORS and CRT groups, but the TORS group may have an advantage with lower feeding tube dependency, suggesting better swallowing functions. 

TORS has become an increasingly accepted treatment option compared with the standard open surgical approach among surgeons due to better functional outcomes and fewer postoperative complications [32]. For treatments focused on recurrent cancers in the oropharynx, White et al. describe the two-year recurrence-free survival rate for TORS as 74%, while that of the open surgical approach group was 43%. Patients treated with TORS also had a significantly lower utilization of a feeding tube resulting in improved swallowing functions compared to those undergoing open surgery [33]. The improved swallowing after undergoing TORS is important because very few patients undergoing salvage surgery via open surgery for oropharyngeal tumors return to a normal diet and the majority maintain a feeding tube at a mean 14-month follow-up [34].

Ford et al. reported that oropharyngeal SCC patient survival rates across three years following TORS were between 89% and 94%, while that of open surgery was between 73% and 85% [35]. Five years after treatment, Nguyen et al. found that overall survival for TORS versus non-robotic surgery was 84.8% and 80.3%, respectively [36]. However, there is conflicting data with regard to survival when comparing TORS and open surgical approach. Data reported by Roselló et al. and Li et al. reflected no significant difference in survival rates when comparing both approaches [32,37]. Currently, there are no published studies of poorer survival outcomes through the primary treatment of TORS when directly compared to open surgery.

Despite many advantages, TORS is not without limitations in treating oropharyngeal malignancies. TORS is in general contraindicated in cases of tonsillar cancer with a retropharyngeal carotid or when lingual arteries, carotid bulb, or internal carotid arteries are at risk depending on tumor location. Functional constraints also apply; TORS is not advisable if the tumor resection necessitates the removal of more than 50% of the deep tongue base musculature or posterior pharyngeal wall or up to 50% of the tongue base along with the entire epiglottis. It also should not be performed in all T4b cancers or otherwise unresectable diseases. When patients have other comorbidities that prohibit the positioning for TORS or access to the oral cavity such as trismus, TORS is also not indicated [38].

Hypopharyngeal cancers are rare and considered to have one of the worst prognoses of all head and neck cancers. At the time of initial diagnosis, most hypopharyngeal cancers develop lymph node involvement and regional and distant metastases, with 50-70% of patients presenting with N1 disease or worse [39,40]. In a study done by Hall et al., 595 patients with SCC of the hypopharynx were analyzed, and it was found that post-treatment recurrences are common and often appear within the first year with metastases occurring in 50% of the first postoperative recurrences. Hall et al. noted that eventually, 64% of patients died of SCC of the hypopharynx [41]. Therefore, treatment for hypopharyngeal cancers remains a challenge, and a multidisciplinary approach is crucial for optimizing survival and maintaining organ preservation for swallowing and speech function [42].

With the growing emphasis on organ preservation, TORS has been increasingly used for treating hypopharyngeal cancer. TORS was found to be safe, feasible, and capable of accessing the hypopharynx and the larynx by Chan et al. [43]. Similar to oropharyngeal cancers, Durmus et al. found that TORS resections of hypopharyngeal cancers were safe to perform, with no intraoperative complications in any of the cases they have included in the study, and no conversion to open case was required [44]. In terms of survival, a study done in 2018 by Mazerolle et al. reported that in 57 patients who underwent TORS for pyriform sinus SCC, overall survival was 84% at 24 months and 66% at 48 months. Additionally, 93% of the patients were able to achieve oral refeeding around five days postoperatively, and overall, 96% of patients were able to tolerate oral diet. Around 54% of the patients followed in this study received adjuvant therapy including chemotherapy and/or RT, while 46% of the patients did not. However, no statistical significance was observed in functional outcome or survival with different adjuvant therapy statuses [45]. The work done by Park et al. in 2012 found that the three-year overall survival rate and disease-free survival rate were 89% and 84%, respectively, for hypopharyngeal SCC following TORS. Moreover, the functional results showed 96% of the patients with favorable swallowing abilities, although this study did not report the survival and functional outcomes of patients who received adjuvant therapy, which was 60.9% of the patients included, and those who only received surgery separately [46]. The research group later investigated the long-term outcomes of TORS in patients with early- and late-stage hypopharyngeal cancer in a 2017 study. Of the 38 patients, 84% received adjuvant therapies, and no data on survival or swallowing function was reported separately based on the status of adjuvant therapy. The group found the five-year disease-specific survival rate and five-year disease-free survival rate for early-stage hypopharyngeal cancer (stages I and II) at 100% each. Advanced-stage hypopharyngeal cancer (stages III and IV) following TORS had a five-year disease-specific survival rate of 74%, and the five-year disease-free survival rate was 68.8%. Around 76.3% of overall patients displayed favorable swallowing abilities with 2.6% of patients requiring a permanent feeding tube [47]. In another study evaluating the outcomes of TORS in patients with stage III or IV hypopharyngeal SCC, over 90% of patients were able to ingest food orally, suggesting appropriate swallowing functions. This study also included patients requiring adjuvant therapies as well as patients who received surgery alone, but it was reported that adjuvant therapy had no statistically significant impact on survival [48]. The studies conclude that TORS improves survival rates in patients with hypopharyngeal cancer and demonstrates excellent swallowing function recovery.   

Traditionally, hypopharyngeal cancer has been treated with open surgery by a combination of laryngectomy and pharyngectomy followed by reconstruction and postoperative RT, which offers promising oncological outcomes at the cost of poor functional outcomes [49]. When comparing the survival rates in TORS or open surgery for hypopharyngeal cancer, Park et al. found no significant differences in the three-year overall survival rate or disease-free survival rate. However, the group found that the TORS group showed better swallowing function compared to that of the open surgery group. In this study, there was no statistically significant difference in tumor staging between the TORS and the open surgery groups and no difference in the number of patients undergoing adjuvant treatment [50]. The similar oncologic outcome between the surgery techniques and better functional recovery of TORS suggests that TORS is a promising surgical treatment option for patients with hypopharyngeal cancer. 

Like TORS in oropharyngeal cancer treatment, the application of TORS is also limited by the extent of malignancy. In general, patients who are eligible for TORS would have more superficial diseases and do not have bony or cartilaginous involvement of the malignancy [51].

## Conclusions

TORS typically yields survival rates that are on par with or exceed those of other treatment modalities for oropharyngeal cancer and tend to result in improved postoperative swallowing function. While evidence consistently supports the advantages of TORS in oropharyngeal cancer management compared to RT and open surgical resection, studies focusing on hypopharyngeal cancer are less abundant, indicating a gap in the literature. TORS is an innovative surgical technique that continues to evolve, has shown promise, and is gaining recognition as a valid treatment option for hypopharyngeal cancer, demonstrating reduced morbidity and enhanced functional outcomes postoperatively, especially when considering an organ-preserving strategy. The precision of fine motor movements, minimal invasiveness, and fewer tissue disruptions in deep anatomical spaces of TORS may account for these benefits as they eliminate surgical incision recovery and avoid the wide profile of radiotherapy toxicities. However, like many other innovative therapeutic options, TORS has its limits, specifically on the location and the extension of the disease processes and whether access and positioning can be achieved. Clinical decision-making for the treatment of oropharyngeal or hypopharyngeal SCC remains grounded in the TNM staging system and often requires a multimodal approach. To optimize patient outcomes, future research is imperative to establish clear guidelines for patient selection and to refine adjuvant therapy protocols, including refining selection criteria for TORS, improving adjuvant therapy protocols, and expanding beyond swallowing function to include a broader range of quality of life measures. 
